# Effect of the Polishing Procedures on Color Stability and Surface Roughness of Composite Resins

**DOI:** 10.5402/2011/617672

**Published:** 2011-07-11

**Authors:** Vera Lucia Schmitt, Regina Maria Puppin-Rontani, Fabiana Scarparo Naufel, Flávia Pardo Salata Nahsan, Mário Alexandre Coelho Sinhoreti, Wagner Baseggio

**Affiliations:** ^1^Department of Restorative Dentistry, Paraná West State University, Rua Universitária, 1619, Jardim Universitário, 85819-110 Cascavel, PR, Brazil; ^2^Department of Pediatric Dentistry, Piracicaba Dental School, UNICAMP, Av. Limeira, 901, 13414-903 Piracicaba, SP, Brazil; ^3^Bauru School of Dentistry, University of São Paulo, Al. Dr. Octávio Pinheiro Brisolla, 9-75, 17012-901 Bauru, SP, Brazil; ^4^Dental Materials Division, Department of Restorative Dentistry, Piracicaba Dental School, UNICAMP, 13083-970 Campinas, SP, Brazil; ^5^UNIPAR, Paranaense University, Rua Rui Barbosa 611, 85810-240 Cascavel, PR, Brazil

## Abstract

*Objectives*. To evaluate the polishing procedures effect on color stability and surface roughness of composite resins. *Methods*. Specimens were distributed into 6 groups: G1: Filtek Supreme XT + PoGo; G2: Filtek Supreme XT + Sof-Lex; G3: Filtek Supreme XT + no polishing; G4: Amelogen + PoGo; G5: Amelogen + Sof-Lex.; G6: Amelogen + no polishing. Initial color values were evaluated using the CIELab scale. After polishing, surface roughness was evaluated and the specimens were stored in coffee solution at 37°C for 7 days. The final color measurement and roughness were determined. *Results*. Sof-Lex resulted in lower staining. Amelogen showed the highest roughness values than Filtek Supreme on baseline and final evaluations regardless of the polishing technique. Filtek Supreme polished with PoGo showed the lowest roughness values. All groups presented discoloration after storage in coffee solution, regardless of the polishing technique. *Conclusion*. Multiple-step polishing technique provided lower degree of discoloration for both composite resins. The final surface texture is material and technique dependent.

## 1. Introduction

Tooth-colored restorations using resin composites have been widely used in comparison with metallic restorations even for posterior teeth with relative success. Patients and clinicians have defined resin composites as the choice material for aesthetic restorations because of their adequate strength, excellent initial aesthetics, moderate cost compared to ceramics, and adhesion to tooth structure. However, due to intrinsic properties of this type of material, they are prone to staining and wear [1]. 

Surface roughness is the major contributor for extrinsic discoloration of resin composite restorations. This property is closely related to the organic matrix, inorganic filler composition of the composite, and finishing and polishing procedures. Rough surface greater than 0.2 *μ*m provides higher chances of biofilm accumulation, leading to staining and/or discoloration of the restoration's body or margins [2].

Composite resin discoloration can occur by three ways: (I) extrinsic discoloration due to biofilm accumulation on the restoration surface; (II) surface or subsurface changes with slight penetration and reaction of dye agents on the superficial layer of composite resin; (III) intrinsic discoloration due to physic-chemical reactions inside the body of the restoration [3].

Moreover, the matrix structure as well as the features of inorganic fillers have a direct effect on surface smoothness of composite resin restorations and on the staining ability. Hydrophilic matrices are more susceptible to water absorption, dye penetration, and staining than hydrophobic ones. Similarly, the filler type and size (glass, pyrogen silicon, and others) are also closely related to staining [4].

In order to measure objectively the color alterations on composite resin restorations, some methods have been experienced, among them the spectrophotometry, which makes the study of several parameters related to color stability of composite resins possible. Vita Easyshade spectrophotometer (Vident, Brea, CA, USA) can measure special sections of visible light spectrum based on the body light reflexion of wavelength specifics. By this method reflected wavelength by a body is changed in values expressed in ΔE* units. The ΔE* values can be used in order to represent the color alterations provided by the composite resin after treatment or period of time [5]. 

In order to provide color stability, wear resistance, and surface smoothness, inorganic fillers have been changed in size and shape [1, 6]. During mastication, wear leads to dislodgment of filler particles. Due to dislodged particles, holes are present on the surface of restoration exposing the organic matrix to oral environment. In addition, these dislodged particles might cause more abrasion to the restoration. Also, the larger and harder the filler, the more wear and degradation might be observed [7].

Nanotechnology has recently been used on composite resin production. The new material represents an evolution on balance of aesthetics and mechanical properties, allowing them to be used in anterior and posterior restorations. Among the advantages of using this material, the following can be pointed out: lower polymerization shrinkage, improved mechanical properties, favoured optical behaviour, greater brightness, surface smoothness, better color stability, and decreased wear [8, 9].

However, not only the material type and composition are responsible for maintaining the smoothness but also the finishing and polishing procedures. These procedures require a sequential using of less abrasive instruments, favouring a smoothness and brightness surface [10]. In order to carry out those procedures, some sets of highly flexible discs polyurethane based and impregnated with aluminum oxide have been used [6]. However, recently, there have been marketed abrasive silicon rubbers in order to provide a smoothness and brightness surface on composite resins. Indeed, the time and clinical steps were reduced [11]. The manufacturers call these systems as one-step systems, since they use only one device [11–13]. However, there are no consensus in the literature concerning the effectiveness of different finishing and polishing procedures and systems used to finish and polish composite resins. Once, some studies have demonstrated that the main procedure to reach adequate smoothness on composite resin surface using the multilayered burs before the using of discs or abrasive rubbers [14]; others reported that the one-step polishing systems are effective [11–13].

In this way, it is important to evaluate the effect of different polishing systems on different composite resin, concerning the roughness and the surface roughness of composite resin and color maintenance on time. 

Therefore, the tested hypothesis at the present study is that composite resin with different filler types submitted to different types of polishing procedures produces different results of surface roughness and staining. The objective was to evaluate the effect of the polishing procedures, single- and multiple-step systems on (1) the color stability and (2) the surface roughness of a nanofilled and a microhybrid composite resin submitted to storage in coffee solution for 7 days.

## 2. Materials and Methods

The manufacturers and the composition of tested composite resins and polishing systems are presented in Table 1.

### 2.1. Specimen Preparation

Cylindrical specimens (7 mm in diameter and 2 mm in height) were fabricated for each group (*n* = 10), according to composite resin and polishing procedures. The composite resin was inserted in the metallic matrix and covered with clear strip and pushed with a glass plate. The specimen was then light cured following the manufacturer's instructions using a halogen light system (Optilux 501, Kerr Corp., Orange, CA.). The curing tip was positioned perpendicular to specimens' surface. The power output density used was 620 mW/cm^2^, frequently monitored by means of a radiometer. The specimens were stored at 37°C, immersed in water distilled for 24 h before the first testing.  Table 2 presents the groups' distribution.

### 2.2. Baseline Color Evaluation

The color of specimens was measured at baseline with a VITA Easyshade (Vident, Brea, CA, USA) spectrophotometer, using the CIELAB scale and the L*, a*, and b*. ΔE* was determined using the following equation: ΔE* = [(Δ*L*)^2^ + (Δ*a*)^2^ + (Δ*b*)^2^]^1/2^. The measurement was performed three times for each specimen. The device was calibrated after the measurement of each specimen.

The specimens were submitted to different polishing systems and procedures, strictly following the manufacturer's instructions. In order to reduce the technique variability, only one operator performed this step. 

After the polishing procedure, each specimen was evaluated according to surface roughness using the Surf-Corder (Kosaka Lab. SE 1700) and Ra as a parameter.

Following the baseline measurements, the specimens were immersed in coffee solution (Nescafe, Nestlé, Switzerland, Batch—91591210B) for 7 days. The coffee solution was the choice as it is one of the most consumed drinks in Brazil and worldwide. Fifteen grams of powder coffee were added to 500 mL boiled water and filtered after 10 min. The coffee manufacturer states that the average time for consumption of one cup of a drink is 15 min, and, among coffee drinkers, the average consumption of coffee is 3.2 cups per day. Therefore, the 7-day storage time simulated 10.080 minutes of consumption of the drink over about seven-month period. The solution was then inserted in the eppendorfs with the specimens [13] and daily renovated.

Before the color was measured, the specimens were washed in distilled water for 1 min and dried with tissue paper. The final color of all specimens was measured as described for the baseline. The effects of discoloration are expressed in ΔE* units and calculated from the ΔL*, Δa*, andΔb* averages using the following equation: ΔE* = [(Δ*L*_0_* − Δ*L*_1_*)^2^ + (Δ*a*_0_* − Δ*a*_1_*)^2^ + (Δ*b*_0_* − Δ*b*_1_*)^2^]^1/2^.

According to Lee et al., 2007, [15]  ΔE* < 1 relates to color alterations not detected by human eye; ΔE* < 3.3—clinically acceptable color alterations; ΔE* > 3.3—clinically not acceptable color alterations, resulting in need of restoration replacement due to aesthetics.

The data were submitted to two-way ANOVA and Tukey's tests with significance level at 5%.

## 3. Results

**Figure 1:**
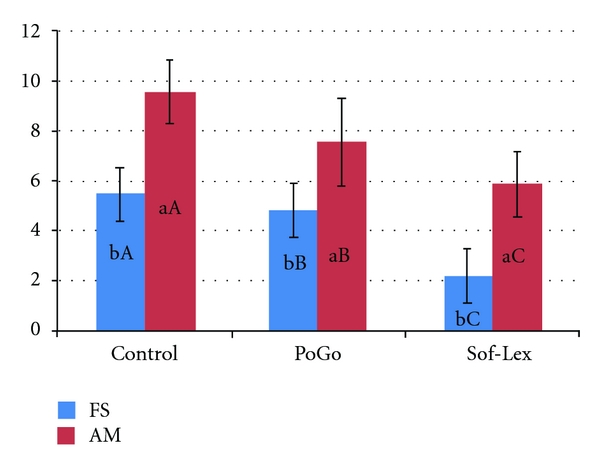
Average and standard deviation values concerning Delta E regarding composite resins (Filtek Supreme (FS) and Amelogen (AM), polishing procedures and coffee storage time (baseline and 7-days storage)). Different small letters mean statistically significant difference between resin composites (*P* > 0.05); different capital letters mean statistically significant difference between polishing procedures (*P* > 0.05).

ANOVA factorial test determined a significant interaction between the studied factors (composite resin and polishing procedures—*P* < 0.01). After 7 days of storage in coffee solution, greater values of color changes for Amelogen were observed, regardless of the polishing procedure (Figure 1).

The higher color changes were observed when Amelogen was not polished compared to the other treatments and composite resins. Filtek Supreme XT showed low color alteration, and the best results were found when it was polished with Sof-Lex. Control groups (3 and 6) showed the highest level of staining, although there was no statistical difference when Filtek Supreme XT was polished with PoGo. 

**Figure 2:**
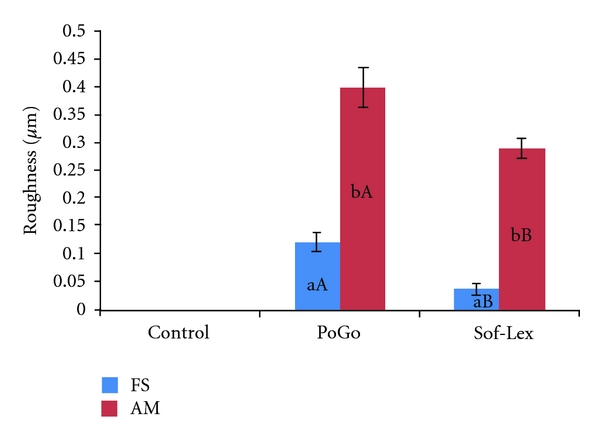
Average values between baseline and final evaluation of surface roughness concerning polishing procedures and composite resins. Different small letters mean statistically significant difference between resin composites (*P* > 0.05); different capital letters mean statistically significant difference between polishing procedures (*P* > 0.05).

Immersion in coffee solution provided higher roughness values for Filtek XT polished with PoGo, while, for Amelogen, the highest values were observed for the nonpolished specimens. Concerning polishing procedures, coffee solution provided lower roughness values for both resins, Sof-Lex polished samples (Figure 2).

## 4. Discussion

Changes in color of composite resins provided by extrinsic factors are attributed to contamination like coffee, tea, nicotine, and beverages. Low periods of immersion, like 7 days, are sufficient to produce staining and color changes to composite resins [13, 16, 17].

Quantitative evaluation of minimal color change by means of visual assessment is not possible or even useful most of times, beside presenting low reproducibility. However, standardized devices can be used for such measurements. Evolution in electronic optics and informatics makes the electronic techniques for color selection more adequate for daily usage [13, 17, 18]. By this reason, in this study the VITA Easyshade system (Vident, Brea, CA, USA) was used. This spectrophotometer measures precise sections of the visible light spectrum, within 400 to 700 nm, based on the reflection of specific body wavelengths, and translating them in values expressed in ΔE* units. These systems are more precise, according to the literature, in comparison with measurements obtained from colorimeters, once they are not influenced by the environment luminosity. ΔE* values can be used to represent color alterations of restorative materials undergoing determined treatment or certain periods of time [5, 13, 17].

The hypotheses tested in this study were accepted. Different polishing procedure systems and different composite, resin produced different levels of polishing and different staining after 7 days in coffee solution immersion. 

Concerning ΔE* value, the use of clear (polyester) strips for nanofilled (Filtek Supreme) and microhybrid (Amelogen plus) composites, resulted ingreater staining values, since resin matrix emerges to the surface, [11–13] which is highly rich in organic components [13, 17, 19]. Moreover, resin matrices tend to absorb more water and are more prone to staining, once water is the vehicle for dyes penetration [13, 17, 19]. 

It was also observed for both composite resins a decrease in ΔE* values in relation to the polishing procedures. The highest values were observed when no polishing was performed, followed by one-step polishing and finally the multiple-step polishing. ΔE* values observed in the present study revealed that the lower the roughness after polishing, the greater the resistance to staining of the composite resins. In the present study, the microhybrid resin composites were the smoothest surfaces against matrix [6, 19]. These surfaces against matrix were smoother than polished surfaces because the unpolished surfaces are composed of more polymer matrix than fillers.

Different polishing methods of finishing a direct composite resin restoration influence the resistance to color and brightness alterations of the restoration [20]. It is clinically important to determine the procedures to be used in order to obtain a smooth surface, with lower time and number of used instruments [17]. This study used single- or multiple-step techniques, and it could be observed that the size and geometry of particles exert a direct impact on the surface smoothness and staining resistance [13]. The combination of nanofillers in nanocluster formulations reduces the interstitial space among fillers, increasing the filler percentage and improving the physical properties. The increase in polishing maintenance in comparison with composites presenting only nanoclusters is also observed, justifying the lowest roughness values for nanofilled composites observed in the present study.

The lowest surface roughness values were observed for the nanofilled composite when the multisteps polishing technique was used. A possible explanation for this observation is the composition and way of usage of the aluminum discs. As they were used in decreasing abrasiveness level, they promote uniform wear and whatever polishing of the surface, regardless of the type of composite resin. However, for the nanofilled composites, this effect is increased once the wear occurs due to individual breakdown of the nanofillers, preserving the nanoclusters [14, 21]. The preservation of nanoclusters is possible as function of the strong chemical interaction between nanocluster and resin matrix [8].

Staining is directly related to the resin phase of composites [13]. Urethane dimethacrylate (UDMA) seems to be more stain resistant than Bis-GMA [18]. However, the resin system of Filtek Supreme consists primarily of Bis-GMA, UDMA, and Bis-EMA. In these restorative systems, the majority of TEGDMA, a somewhat hydrophilic monomer, has been replaced by a blend of UDMA and Bis-EMA [7]. According to the manufacturers, Filtek Supreme composite resins impart a greater hydrophobicity to the composite resin. The low staining susceptibility of Filtek Supreme may be related to a low water sorption rate due to the use of hydrophobic resins [18].

The multiple-step technique demonstrated to be most effective in obtaining a smoother surface, even for the microhybrid composite resin. This fact can be explained by the operationalization of using these materials, as they are usually structured in sequential order of using with abrasiveness decreasing, favoring the final surface texture. This scenario does not occur with the one-step materials [1, 17].

Studies report that aluminum oxide flexible discs are the best instruments to generate low roughness in resin surfaces. Lu et al. [15], Türkün et al. [17], and Venturini et al. [22], demonstrated that aluminum oxide discs are capable of providing smooth surfaces, and this fact is related to their capacity to reduce fillers and matrix evenly. This justifies that the multiple-step systems evaluated during the present study were more effective in providing smoother surfaces for both, microhybrid and nanofilled composites [23]. The present results corroborate with those found by Watanabe et al. [24], who demonstrated that surface finishing with multiple steps systems was superior to one-step systems. 

The single-step system, PoGo, was used in the present study with no surface pretreatment. This system presented higher surface roughness values in comparison with the Sof-Lex discs, regardless of the evaluated composite resin. Similar results were obtained by Yap et al. [6]. Although the manufacturer recommends the use of the Enhance system prior to PoGo, Jung et al. [23], while evaluating the surface of microhybrid and nanofilled composite resins, polished with Enhance/PoGo association, observed no beneficial results on the composite surface quality with the pretreatment with the Enhance system. 

On the other hand, Türkün et al. [17], investigated the surface roughness of microhybrid and nanofilled composite resins when polished with Sof-Lex discs and the PoGo system. They used medium, fine, and ultrafine Sof-Lex discs for 30 seconds each for each of the composite resin samples and the PoGo discs for 30 seconds, using a light rotation movement. They observed that PoGo system promoted a smooth finishing for all the samples in a shorter period of time in comparison to the Sof-Lex discs and revealed that PoGo system saves time in comparison to multiple-step systems. The multiple-step systems provided the greater staining resistance for both, the nanofilled and microhybrid composite resins.

## 5. Conclusion

Based on the results of the present study, it was concluded that the multiple-step polishing technique promoted greater staining resistance, for both the nanofilled and microhybrid composites, and provided the lowest values of surface roughness. 

The final surface texture is material and technique dependent. The best results for roughness and staining resistance were obtained from the association of nanofilled composites and multiple-step polishing procedures.

## Figures and Tables

**Table 1:** Composition and manufacturers of the materials used in the study.

Material (Shade)	Manufacturer	Composition	Filler average	Filler loading
Filtek Supreme XT (A2)	3M ESPE, St Paul, MN, USA	Matrix*:* Bis-GMA, UDMA, Bis-EMA, and TEGDMA Filler*:* (zirconia/silica)	Nanofillers of silicon (5–75 nm), zircon/silicon nanoclusters (0.6–1.4 *μ*m)—nanofiller	78.5% wt, 59.5% vol

Amelogen plus (A2)	Ultradent Inc., South Jordan, UT, USA	Matrix*:* Bis-GMA and diluent Filler*:* silicon dioxide, silicon, silicate particles	Microhybrid	76% w, 61% v

PoGo	Dentsply Caulk, Milford, DE, USA	Cured composite of urethane dimethacrylate, fine diamond powder, silicon dioxide 7 *μ*m, Al_2_O_3 _		

Sof-Lex discs	3M ESPE Dental Products, St. Paul, MN, USA	Al_2_O_3_ flexible discs 29 *μ*m (M) 14 *μ*m (F) 5 *μ*m (SF)		

**Table 2:** Groups distribution according to composite resin and polishing procedure.

Group	Composite resin + treatments
1	Filtek Supreme XT + PoGo
2	Filtek Supreme XT + Sof-Lex pop on
3	Filtek Supreme XT + mylar strip
4	Amelogen + PoGo
5	Amelogen + Sof-Lex pop on
6	Amelogen + mylar strip
